# Parents of minor children lose less weight during a behavioral weight loss intervention: Findings from the Rural LEAP trial

**DOI:** 10.1002/osp4.604

**Published:** 2022-04-07

**Authors:** Christie A. Befort, Kathryn M. Ross, David M. Janicke, Michael G. Perri

**Affiliations:** ^1^ Department of Population Health University of Kansas Medical Center Kansas City Kansas USA; ^2^ Department of Clinical and Health Psychology University of Florida Gainesville Florida USA

**Keywords:** behavior therapy, parents, weight loss

## Abstract

**Objective:**

The transition to parenthood is associated with worsening health behaviors, yet the impact of parental status on successful weight loss has rarely been examined. The purpose of this study was to examine the effect of parental status of minor children on weight loss and behavioral adherence in a rural community‐based weight loss intervention.

**Methods:**

Five hundred and twenty‐eight adults (age 21–75 years, body mass index [BMI] 30–45 kg/m^2^) were enrolled in a group‐based weight loss intervention consisting of 16 weekly sessions delivered in face‐to‐face group sessions at Cooperative Extension Service (CES) offices. Participants who were parents with at least one minor child (≤18 years old) in the home were compared to participants with no minor children in the home. Measures included percent weight loss, session attendance, adherence to self‐monitoring, and achieving calorie and physical activity goals.

**Results:**

Compared to participants without minor children, parents with minor children lost significantly less weight (7.5% vs. 6.2%, respectively; *p* = 0.01), and were less likely to lose ≥5% of baseline weight (59.2% vs. 70.2%, respectively; *p* = 0.02). In addition, parents with minor children attended significantly fewer sessions, had lower adherence to self‐monitoring, and met calorie and step goals less often (all *p*s < 0.001). The association between parental status and percent weight loss was not significantly moderated by gender of the parent.

**Conclusions:**

Parents of minor children had greater difficulty adhering to intervention goals and lost less weight than participants without minor children. Future research should investigate whether tailoring intervention to meet the unique needs of parents can enhance outcomes, especially given the large segment of the population represented by this group.

## 1 INTRODUCTION

The transition to parenthood has been found to be associated with worsening health behaviors including decreases in physical activity,^1^, ^2^ increases in unhealthy eating,^3^ and weight gain.^4^ This “child effect” appears to be sustained throughout child‐rearing years^4^, ^5^ and may be related to greater stress, time‐related barriers, and changes in the home food environment.^6^, ^7^, ^8^ Despite this, parental status has rarely been studied as an important factor influencing successful weight loss.

The extent to which parental status influences one's ability to change health behaviors may vary across geographical and sociocultural contexts. Compared to urban settings, rural areas have a higher prevalence of obesity,^9^, ^10^ fewer resources to support healthy lifestyles,^11^, ^12^ and reduced access to evidence‐based treatment.^13^ Rural culture also has a strong focus on family and community,^14^ and the emphasis on child‐rearing activities may exert some unique influences on parent health behavior change in rural environments. Barriers faced by parents may also differ by the age of their children as well as the number of children in the home. For example, during the elementary age years, busy extracurricular activity schedules and child eating preferences (“picky eating”) may exert distinct barriers to time demands or changing the home food environment.^15^

A recent post‐hoc analysis from an adult behavioral weight loss trial conducted in an urban setting found that men with one or more minor children in the home lost significantly less weight (−8.6%) compared to men without minor children (−11.7%), however this effect was not observed among women (−9.4% vs. −8.5% for women with vs. without minor children, respectively).^16^ However, no studies have examined the effect of parental status on weight loss during a behavioral intervention in a rural setting. In addition, the impact of the developmental status or age of children remains unknown. Thus, the current study aimed to examine the effect of parental status of minor children on weight loss among rural residents who were enrolled in a community‐based behavioral weight loss intervention. A secondary purpose was to examine the effect of parental status on important behavioral measures, including session attendance and adherence to program goals for self‐monitoring, diet, and physical activity. Finally, exploratory analyses examined additional variance accounted for by participant age, whether effects differed by the age or number of minor children, or if the association between having minor children in the home and weight change was moderated by the gender of the parent.

## 2 METHODS

### 2.1 Participants

Participants were adults with obesity (age 21–75 years, body mass index [BMI] 30–45 kg/m^2^) enrolled in the Rural LEAP trial, a randomized controlled trial of three extended‐care weight loss maintenance interventions conducted at Cooperative Extension Service (CES) offices in 14 rural counties in northern Florida.^17^, ^18^ The first phase of the trial occurred prior to randomization and included a 4‐month face‐to‐face, group‐based behavioral weight loss program. During the second phase, participants were randomized to one of three 12‐month extended‐care programs that provided additional intervention delivered via individual telephone sessions, group telephone sessions, or email (an educational control condition). The current study used data from the first 4‐month weight loss phase, conducted prior to trial randomization.

As previously reported,^17^ exclusion criteria for Rural LEAP included the use of medications known to affect body weight, musculoskeletal conditions that precluded walking for 30 min, weight loss >4.5 kg in the preceding 6 months, uncontrolled diabetes or hypertension, and clinically significant depression or substance abuse. A total of 528 participants were enrolled in the initial weight loss phase of the Rural LEAP trial. At baseline, these participants had a mean BMI of 36.6 kg/m^2^, 82% were female, 74% identified as non‐Hispanic White, and 19% identified as non‐Hispanic Black. The study was approved by the University of Florida Institutional Review Board, and all participants provided written informed consent.

### 2.2 Intervention

The weight loss intervention, described in detail previously,^17^ included 16 weekly sessions delivered in face‐to‐face group sessions. Intervention content was modeled after the Diabetes Prevention Program lifestyle intervention,^19^ with adaptions made to specifically address barriers to weight loss experienced by adults living in rural areas. The intervention was not specifically tailored to address barriers related to having children in the home; however, problem‐solving barriers during discussion was a key component and included barriers related to household members. Participants were instructed to self‐monitor caloric intake and physical activity daily, using either study‐provided paper records (combined with a printed calorie reference book and standard pedometer) or digital alternatives (e.g., calorie tracking websites, digital activity monitors owned by participants). Caloric intake goals were based initially on baseline weight (i.e., 1200 kcal per day for individuals weighing <113.6 kg and 1500 kcal per day for individuals weighing ≥113.6 kg) and were subsequently modified based on individual weight loss progress. Physical activity goals focused on walking; initial step goals were based on participants' average daily steps (as assessed during week 2 of the program), with gradual increases of 500–1000 steps/day (∼5–10 min/day of walking) each week until the goal of 6000 steps/day above baseline was met (representing an increase in 60 min/day of walking). Interventionists collected self‐monitoring records at each session and provided feedback regarding potential changes to improve adherence and goal attainment. Participants were given the opportunity to schedule an individual make‐up session if they missed a group session.

### 2.3 Measures

Weight was measured with a calibrated digital scale (Tanita BWB‐800S) with participants in light indoor clothing, pockets emptied, and shoes removed. Number and ages of children and whether they lived in the home were collected at baseline via a self‐report questionnaire assessing family structure and contextual factors.^20^ Adherence to self‐monitoring was measured as percent of days that calorie records and step records were kept, calculated from the self‐monitoring records returned to interventionists. If any calorie information was recorded for any meal during a given day, or if any step count information was recorded, it was scored as a day that the participant kept a calorie record or a step record, respectively. Missing data were assumed to represent a day when self‐monitoring was not completed. Achievement of two goals (caloric intake per day and steps per day) was based on participants' self‐reported daily assessment (yes or no) recorded on the self‐monitoring record for each goal separately. For days without a record or an incomplete record (i.e., missing self‐reported goal achievement or missing type and quantity of food for one meal), it was assumed that the goal was not met.

### 2.4 Analyses

All analyses were conducted in R version 4.0.5. Missing weight data were imputed by carrying baseline observations forward, conservatively assuming that participants who did not return at follow‐up returned to baseline weight. Linear regression models were used to compare percent weight change from baseline to month 4 between participants who had at least one minor child (≤18 years) in the home and those who did not. Non‐parents and parents of only older‐aged children (>18 years) were collapsed into a single comparison group, as has been done previously,^21^ based on the assumption that parents whose children were >18 years were likely to be more similar to non‐parents (e.g., having similar levels of control over their time and home environments as non‐parents). Chi‐square analysis was used to examine differences in the proportion who lost ≥5% between participants who had minor children in the home and those who did not. Due to significant skew, differences in attendance and adherence were assessed using Kruskal–Wallis (KW) tests. Chi‐square analyses and linear regressions were used to examine associations between potential covariates (age, sex, race/ethnicity, education, and employment status) and parental status and percent weight change. Having children in the home is theorized to be one of many factors that may explain previously observed associations between participant age and weight loss outcomes ^22^; thus, the additional variance in weight loss explained by age, after controlling for parental status, was examined using hierarchical regression. Finally, linear regression models were used to assess whether there was an association between number of children in the home and percent weight change and whether percent weight change was moderated by parent gender. Binary variables were created to categorize the age of children in the home using typical schooling categories (i.e., whether or not a participant had a child [or children] age ≤5 years = toddler/preschool; 6–12 years = elementary; 13–16 = middle school/younger high school; and 17–18 = older high school) and were used in a regression to examine whether the age of children in the home was associated with percent weight change.

## 3 RESULTS

Of the 528 participants enrolled in the weight loss phase of the Rural LEAP trial, 142 participants (26.9%) had at least one minor child in the home; of these, 71 had one minor child, 46 had two minor children, and 25 had three or more minor children. The average age of minor children was 11.6 ± 4.9 years. Participants with minor children were younger than participants without minor children (see Table 1); however, there were no other significant differences in baseline characteristics.

**TABLE 1:** Baseline participant characteristics by parental status of minor children

	No minor children (*n* = 386)	≥1 minor child (*n* = 142)	*p* Value
Weight, mean (SD), kg	100.3 (15.6)	101.2 (13.3)	0.54
Body mass index, mean (SD), kg/m^2^	36.6 (3.9)	36.6 (3.6)	0.90
Age, mean (SD), years	58.2 (8.9)	44.5 (8.6)	<0.001
Sex, *n* (%), female	312 (80.8)	122 (85.9)	0.18
Race/ethnicity, *n* (%)			0.10
White, not Hispanic/Latino	290 (75.1)	101 (71.1)	
Black, not Hispanic/Latino	73 (18.9)	27 (19.0)	
Other, not Hispanic/Latino	11 (2.8)	5 (3.5)	
White, Hispanic/Latino	8 (2.1)	8 (5.6)	
Black, Hispanic/Latino	0 (0.0)	1 (0.7)	
Other, Hispanic/Latino	4 (1.0)	0 (0.0)	
Education level, *n* (%)			0.89
<High school	9 (2.3)	3 (2.1)	
High school or GED	201 (52.1)	79 (55.6)	
Associate's degree	47 (12.2)	19 (13.4)	
Bachelor's degree	88 (22.8)	29 (20.4)	
Advanced degree	41 (10.6)	12 (8.5)	
Annual household income			0.31
<$20,000	46 (11.9)	21 (14.8)	
$20,000–$34,999	64 (16.6)	29 (20.4)	
$35,000–$49,999	63 (16.3)	30 (21.1)	
$50,000–$74,999	96 (24.9)	27 (19.0)	
>$75,000	91 (23.6)	29 (20.4)	
Unknown/refused	26 (6.7)	6 (4.2)	
Have minor children, by age of child, *n* (%)			
≤5 years old	–	26 (18.3)	
6–12 years old	–	65 (45.8)	
13–16 years old	–	63 (44.4)	
17–18 years old	–	40 (28.2)	

**FIGURE 1:**
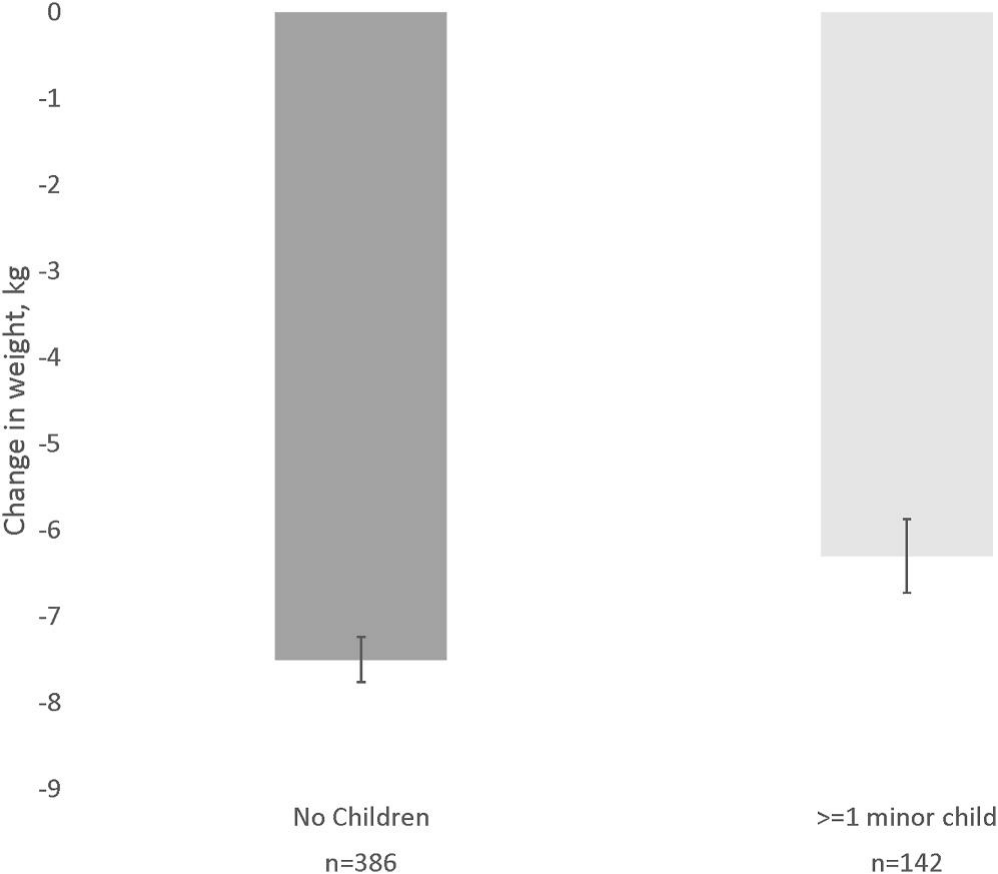
Mean weight loss (kg) by parental status of minor children. Error bars represent standard errors

Overall retention at month 4 was 88.1%, and there was no significant difference in retention by parental status. On average, participants lost 7.17 ± 5.38 kg from baseline to month 4, representing a 7.16 ± 5.13% change from baseline weight. There was a significant difference in weight loss depending on the presence of minor children in the home, *F*(1,526) = 6.70, *p* = 0.01, such that participants with minor children lost significantly less weight (−6.21 ± 5.10%) compared to those without minor children (−7.50 ± 5.10%); see Figure 1. Moreover, participants with minor children were significantly less likely to achieve weight losses ≥5% from baseline compared to those without minor children (59.2% vs. 70.2%, respectively; *Χ* ^2^(1) = 3.31, *p* = 0.02).

Table 2 shows results for adherence measures. Participants with minor children demonstrated significantly lower session attendance when attendance was calculated both without makeup sessions (KW *Χ* ^2^(1) = 12.12, *p* < 0.001) and with makeup sessions (KW *Χ* ^2^(1) = 8.79, *p* = 0.003). Moreover, there were significant differences in all adherence outcomes, such that participants with minor children demonstrated lower adherence to keeping calorie records (KW *Χ* ^2^(1) = 18.33, *p* < 0.001), meeting calorie goals (KW *Χ* ^2^(1) = 17.79, *p* < 0.001), keeping step records (KW *Χ* ^2^(1) = 12.56, *p* < 0.001), and meeting step goals (KW *Χ* ^2^(1) = 13.88, *p* < 0.001).

**TABLE 2:** Program adherence by parental status of minor children

	No minor children (*n* = 386)	≥1 minor child (*n* = 142)	*p* Value
Attendance, mean (SD)			
Percent attendance without makeups	81.8 (21.7)	75.3 (24.2)	<0.001
Percent attendance with makeups	89.6 (21.1)	84.5 (24.7)	0.003
Adherence, percent of days, mean (SD)			
Calorie records kept	86.7 (24.2)	77.0 (28.9)	<0.001
Calorie goals met	61.2 (25.6)	50.5 (26.5)	<0.001
Step records kept	81.4 (26.5)	71.5 (30.5)	<0.001
Step goals met	49.8 (25.4)	40.8 (22.9)	<0.001

Only age significantly differed by parental status, therefore age was the only variable considered as a possible covariate in the association between minor children in the home and percent weight change. Older age was associated with significantly greater weight loss (*F*(1,526) = 11.57, *R* ^2^ = 0.02, *p* < 0.001), such that each additional year of age was associated with a −0.07 ± 0.02% greater weight loss. After controlling for parental status, age explained an additional 1% of variance in weight loss outcomes (*F*(1,525) = 5.48, *R* ^2^_change_ = 0.01, *p* = 0.02). Although sample size precluded statistical analyses, visual inspection of means in only adults aged 40–59 demonstrated a similar pattern as the primary outcome analysis, such that participants with minor children in the home lost less weight than those without (−6.45 ± 4.80% vs. −7.26 ± 5.19%, respectfully).

The association between children in the home and percent weight change was not significantly moderated by gender of the parent, *p* = 0.74. There were also no statistically significant differences in weight change by the number of children in the home, *p* = 0.06. The overall model showed no significant difference by the age of children in the home, *p* = 0.28, however there was some indication that parents with a child in the 6–12 age range lost less weight (−5.87 ± 4.62%) compared to those with no minor children (−7.50 ± 5.10%; *p* = 0.05).

## 4 DISCUSSION

The major finding of this study was that, in a rural community‐based weight loss intervention, participants with minor children in the home lost significantly less weight compared to participants without minor children in the home. The difference was meaningful in magnitude (6.2% vs. 7.5% weight loss), and a significantly smaller proportion of participants with minor children achieved the clinical threshold of ≥5% loss of baseline weight (59.2% vs. 70.2%). Importantly, findings support the idea that the absence of children in the home may be one of several reasons why adults ≥60‐years‐old have been observed to lose more weight.^22^ With 40% of family households in the United States having minor children,^23^ parental status is highly common and important characteristic to consider when designing weight loss interventions to improve the population‐level health benefits.

Results of the current study also identified potential mechanisms by which parents of minor children may have poorer weight loss outcomes. Participants with minor children performed significantly worse on key indicators of intervention adherence (session attendance, self‐monitoring, and meeting diet and physical activity goals), all well‐established mediators of weight loss.^24^, ^25^ The mechanisms by which parenting roles can influence attempts at behavior change are complex,^26^, ^27^ and may include competing demands (e.g., related to caretaking), stress and time‐related barriers between work and family roles,^28^ and the influence of children, family dynamics, and the home environment on food choices and screen time.^29^, ^30^, ^31^ In addition, parents of minor children may differ from non‐parents or parents of older children in other ways that indirectly influence weight loss success, for example, differences in living situations, neighborhood contextual factors, or prioritization of weight and health behaviors. The current study found that both diet and physical activity adherence were impacted by parental status to a similar magnitude (approximately 10% lower adherence), and that session attendance was lower even with opportunities for individually scheduled make‐up sessions. It is likely that multiple strategies will be needed to address the variety of unique factors for parents of minor children. Additional research is needed to understand individual‐, family‐, and environmental‐level factors influencing parents' ability to successfully adhere to weight loss behaviors.

There have been very few adult weight loss interventions targeted specifically for parents, and these have been limited to mothers with infant to preschool age children.^32^ This study found less weight loss for parents across both men and women, contrary to one prior study that observed the effect for men only.^21^ In addition, findings indicated that parents with elementary age children may experience the most barriers. Caretaking demands may interfere more when parents are accommodating children's own choices and activities (e.g., food preferences and extracurricular activities) while the children lack the greater independence of older children (e.g., own transportation and food preparation). These findings highlight the need to develop interventions that meet the needs of both women and men who have minor children inclusive of an elementary age range.

Weight loss interventions designed for parents not only have potential to benefit a large segment of the population, but also hold potential to produce a ripple effect on the minor children of parents enrolled in these programs. Obesity runs in families through hereditary components, home environment, family functioning, and behavior modeling.^33^ Likewise, parents' own health behavior change and successful weight loss is positively related to their children's health behavior change and weight loss.^34^

This study has several limitations. Sample size restrictions limited the ability to further disentangle the effects of age versus parental status of minor children (e.g., by examining the effect of parental status within subgroups of participants of more narrow age ranges), as well as to test for mediation of the observed effects by adherence variables, or whether adherence differed by the ages of children in the home. Data were not available to examine the potential role of different family structures (e.g., single parent vs. two parent households). The sample consisted predominantly of White non‐Hispanic women; men were under‐represented as is common in behavioral weight loss trials.^35^ In addition, participants with minor children who enrolled in the study may not represent the broader population of parents with minor children (e.g., in terms of motivation, resources, and time commitments). Lastly, the study was conducted in a rural CES setting that emphasizes child development and family opportunities (e.g., 4‐H), and findings may not generalize to other types of settings. Strengths of the study include the relatively large sample in an underserved rural setting, the inclusion of multiple adherence measures, and the pre‐randomized intervention that eliminates any confounding from different treatments.

## 5 CONCLUSION

Participants with minor children in the home lost significantly less weight, attended fewer sessions, and performed significantly worse on self‐monitoring and attainment of behavioral goals. Novel interventions are needed to address barriers unique to parents.

## CONFLICT OF INTEREST

The authors have no conflicts of interest to disclose. Drs. Befort, Ross, Janicke, and Perri report grants from the National Institutes of Health during the conduct of the study.

## CLINICAL TRIAL REGISTRATION

ClinitalTrials.gov Identifier: NCT02054624.

## AUTHOR CONTRIBUTIONS

Michael G. Perri, Christie A. Befort, Kathryn M. Ross, and David M. Janicke conceived and carried out the trial. Kathryn M. Ross conducted data analyses. All authors were involved in writing the paper and had final approval of the submitted and published versions.
